# *In vivo* functional and morphological characterization of bone and striated muscle microcirculation in NSG mice

**DOI:** 10.1371/journal.pone.0183186

**Published:** 2017-08-11

**Authors:** Haider Mussawy, Lennart Viezens, Gerrit Hauenherm, Malte Schroeder, Christian Schaefer

**Affiliations:** 1 Department of Orthopaedic Surgery, University Medical Centre Hamburg-Eppendorf, Hamburg, Germany; 2 Department of Trauma-, Hand-, and Reconstructive Surgery, University Medical Centre Hamburg-Eppendorf, Hamburg, Germany; 3 Department of Spine Surgery, Klinikum Bad Bramstedt, Bad Bramstedt, Germany; University of Arizona, UNITED STATES

## Abstract

Organ-specific microcirculation plays a central role in tumor growth, tumor cell homing, tissue engineering, and wound healing. Mouse models are widely used to study these processes; however, these mouse strains often possess unique microhemodynamic parameters, making it difficult to directly compare experiments. The full functional characterization of bone and striated muscle microcirculatory parameters in non-obese diabetic-severe combined immunodeficiency/y-chain; NOD-Prkds IL2rg (NSG) mice has not yet been reported. Here, we established either a dorsal skinfold chamber or femur window in NSG mice (n = 23), allowing direct analysis of microcirculatory parameters *in vivo* by intravital fluorescence microscopy at 7, 14, 21, and 28 days after chamber preparation. Organ-specific differences were observed. Bone had a significantly lower vessel density but a higher vessel diameter than striated muscle. Bone also showed higher effective vascular permeability than striated muscle. The centerline velocity values were similar in the femur window and dorsal skinfold chamber, with a higher volumetric blood flow in bone. Interestingly, bone and striated muscle showed similar tissue perfusion rates. Knowledge of physiological microhemodynamic values of bone and striated muscle in NSG mice makes it possible to analyze pathophysiological processes at these anatomic sites, such as tumor growth, tumor metastasis, and tumor microcirculation, as well as the response to therapeutic agents.

## Introduction

NSG mice (non-obese diabetic-severe combined immunodeficiency/y-chain; NOD-Prkds IL2rg) combine a severe immune deficiency mutation [SCID] and IL-2 receptor y-chain deficiency. This y-chain is a crucial component of the high-affinity receptors for IL-2, IL-4, IL-7, IL-9, IL-15, and IL-21. The absence of the IL-2R y-chain leads to severe impairments in T- and B-cell development and function, and completely prevents NK-cell development. These mice exhibit an extended lifespan and show superior and sustained engraftment of human hematopoietic stem cells and human malignant cells compared to other immunodeficiency mice, such as NOD.CB17-Prkdc^scid^, (NS)-related mice and NOD.Cg-Prkdc^scid^B2m^tm1Unc^/J, and (NSB)-related mice [1–3]. This makes NSG mice a valuable tool in neoplastic research.

The organ-specific microenvironment is strongly linked to angiogenesis and microcirculation, which play key roles in processes such as wound and fracture healing, organ-specific tumor growth, and metastasis. To further elucidate these processes, a full characterization of microcirculatory parameters is essential. To our best knowledge, there are no published studies evaluating these microcirculatory parameters *in vivo* in this mouse model.

The dorsal skinfold chamber model allows repetitive visualization of morphology and hemodynamics in different regions of interest (ROIs) in striated muscle over time by intravital fluorescence microscopy. This model has demonstrated feasibility and reproducibility in several published experimental studies in the mouse, rat, and hamster [4–7]. To analyze bone microcirculation *in vivo* under physiologic and pathologic situations with repetitive intravital microscopy, Hansen-Algenstaedt et al. [8] developed the femur window in 2005. In subsequent studies, this chamber model was used to analyze the bone tumor microenvironment [9–11]. Here, we aimed to present the first comparative characterization of the microcirculatory properties of bone and striated muscle in NSG mice over a period of 28 days. Furthermore we aimed to compare the NSG mouse strain to other mouse strains in terms of microhemodynamic parameters.

## Animals, materials, and methods

### Animals

For our study, we used 12- to 14-week-old male NSG mice (non-obese diabetic-severe combined immunodeficiency/y-chain; NOD-Prkds IL2rg; University Medical Centre Hamburg-Eppendorf, Germany). The experiments were in respect of Replacement, Refinement and Reduction (3R) with defined animals per group and lower pain/distress for the animals. For this reason, all animals received Metamizole (20 mg/kg) dissolved in the drinking water for analgesia. During the experiments, the animals were housed individually (one animal per cage) on a 12:12 h light:dark cycle and had free access to tap water and standard pellet food (Altromin; Lage, Germany). The animals were monitored two to three times daily post-operatively by trained animal keepers for sleeping habits, feeding habits, grooming behavior (whether each mouse wiped its fur, ears, tail, and genitals), and space use (whether each mouse used the whole space available in the cage). Animals were weighed upon arrival and weekly thereafter. The animal room was restricted to the use of NSG mice. Clinical signs of postoperative infections, damage to the chamber or loss of greater or equal as 15% of the initial weight were defined as exclusion criteria.

The study was approved by the local governmental animal care committee (protocol number 05/12) and was conducted in accordance with German legislation on the protection of animals and the NIH Guidelines for Care and Use of Laboratory Animals (NIH Publication #85–23 Rev. 1985).

#### Preparation of the dorsal skinfold chamber

**Fig 1:**
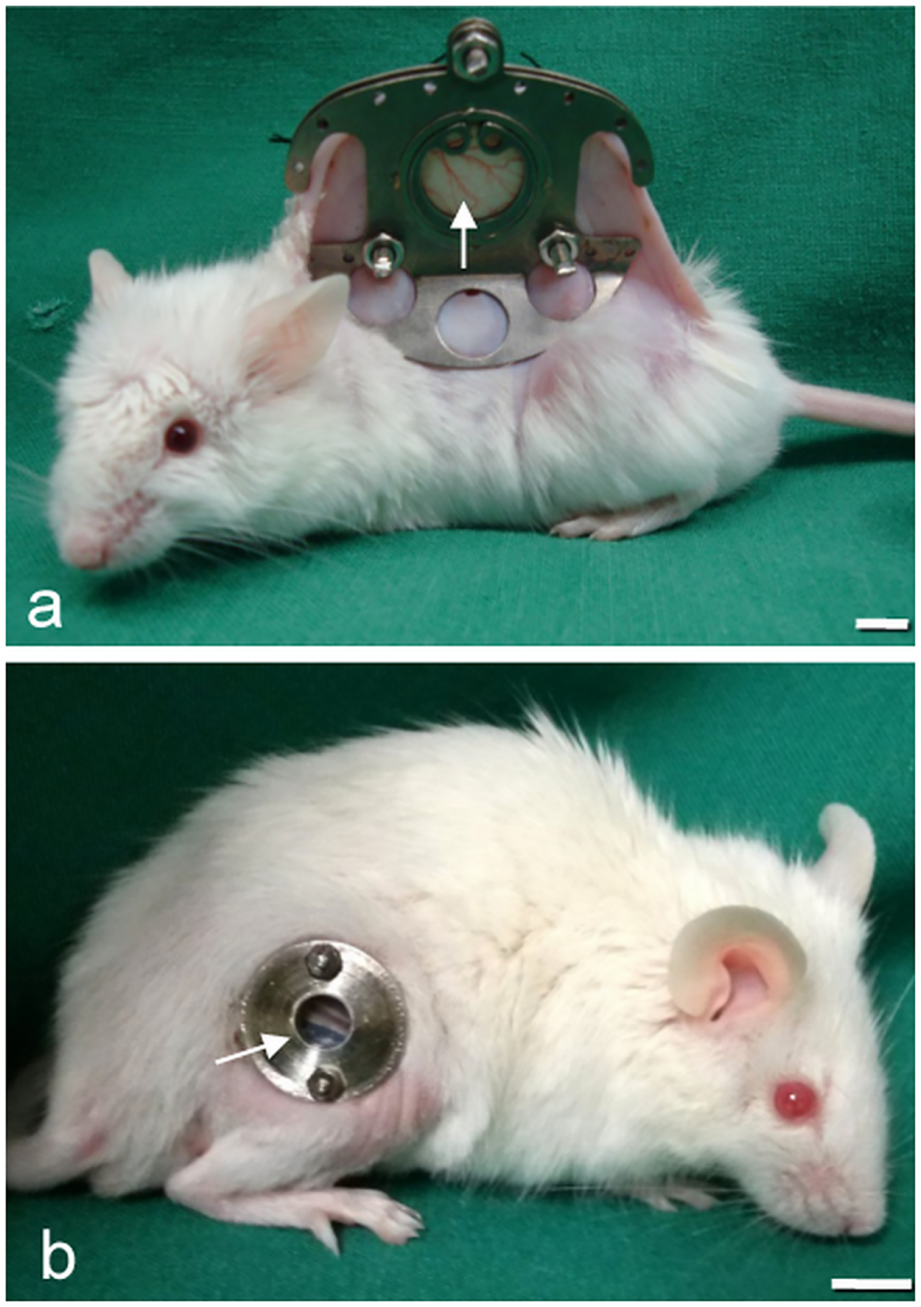
NSG mouse with chamber implantation. (a) NSG mouse equipped with a dorsal skinfold chamber (DSC) or (b) a femur window (FW) and observation window (arrow), showing that the microcirculation of the striated muscle and bone can be analyzed by intravital fluorescence microscopy. (scale bars a-b = 7 mm).

The microcirculation of striated muscle was analyzed within the dorsal skinfold chamber model (n = 11; Fig 1a).

**Fig 2:**
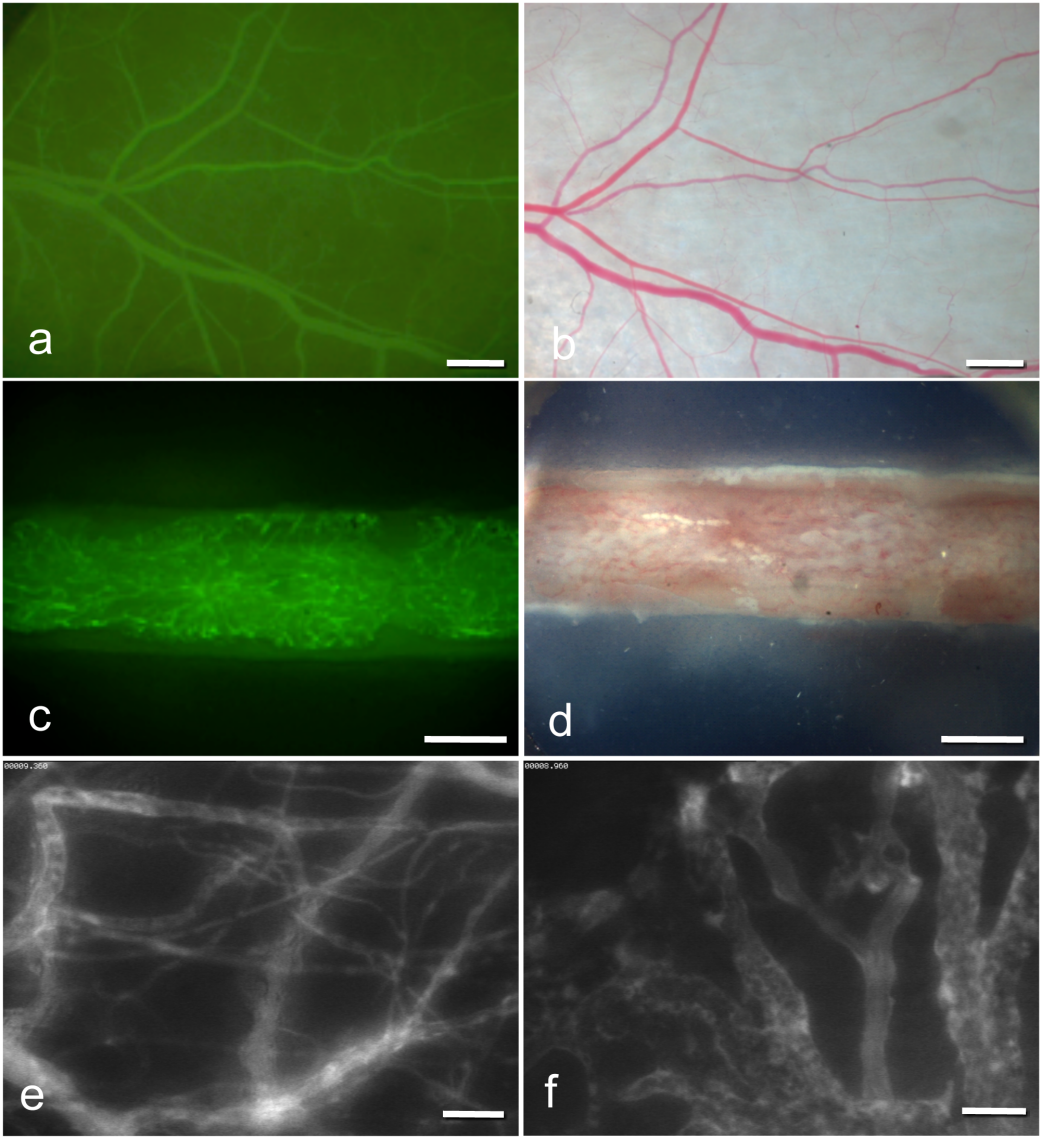
Intravital fluorescence microscopy (IVM) images. (a) IVM of microvessels in the dorsal skinfold chamber (1.25× objective) and in the femur window (c) (2.5× objective) after contrast enhancement by 0.1 ml of 5% FITC-labeled dextran 150.000 i.v. (b;d) Stereo microscopy images of microvessels in the dorsal skinfold chamber (b) and in the femur window (d). (e;f) Intravital fluorescence microscopy of microvessels in the dorsal skinfold chamber (e) and microvessels in the femur window (f) (20× objective) by blue light epi-illumination. (scale bars a-b = 680 μm, c-d = 500 μm, e-f = 70 μm).

The chamber preparation has been described previously in detail [7]. Briefly, NSG mice were anesthetized by intraperitoneal (i.p.) injection of ketamine (75 mg/kg of body weight; Pharmacia, Erlangen, Germany) and xylazine (15 mg/kg of body weight; Rompun, Bayer, Leverkusen, Germany). All surgical procedures were performed under aseptic conditions with sterile instruments and gloves while maintaining body temperature at the physiological level using a heating plate (Omnilab PST 100, Jiirgens, Germany). Subsequently, two symmetrical titanium frames were implanted on the extended dorsal skinfold of the animals, so that they sandwiched the double layer of skin. One layer of skin was completely removed in a circular area of ~15 mm in diameter. The remaining layers, consisting of striated muscle (Fig 2b), subcutaneous tissue, and skin, were covered with a removable cover glass, which was fixed in one of the titanium frames with a snap ring.

#### Preparation of the femur window

The microcirculation of bone was analyzed within the femur window (n = 12; Fig 1b). The chamber preparation has been described previously in detail [8]. The anesthesia, aseptic surgical conditions, and use of heating plates during the operation were the same as described for the dorsal skinfold chamber. Briefly, a ~10 mm longitudinal skin incision was made approaching the femur from the side. The femur was exposed by blunt dissection between the muscles. Subsequently, the corticalis was ground down with a scalpel over an area of 5 × 1 mm to establish a plane of visualization of cancellous bone microvasculature (Fig 2d). Then, a clamp was inserted with medial contact to the femur. The femur window, with the glass coverslip at the bottom, was brought to the side of the visualization plane and fixed to the clamp using two nuts. The empty space within the femur window was filled bilaterally with Ostron Cement (Ostron 100; GC Corporation, Tokyo, Japan) and Gelita^®^ (Braun, Aesculap, Germany) between the cement and bone.

### Intravital fluorescence microscopy

The dorsal skinfold chamber or femur window were attached to the microscopic stage. For contrast enhancement of the microcirculation, 0.1 ml of 5% fluorescein isothiocyanate (FITC)-labeled dextran 150.000 (Molecular Probes, Invitrogen Lld., Paisley, UK) was administered via the tail vein (Fig 2a and 2c). For the measurement of the effective vascular permeability, 0.1 ml of 5% FITC was mixed with bovine serum albumin (FITC-BSA, Molecular Probes, Invitrogen Lld., Paisley, UK) and also administered via the tail vein. Microscopy was performed with a Zeiss Axiotech microscope (Zeiss, Oberkochen, Germany) equipped with a 100 W mercury lamp attached to an epi-illumination filter block for blue, green, and ultraviolet light. The microscopy images were recorded by a charge-coupled device video camera (Hamamatsu C-0377-1, Hamamatsu Photonics, Germany) and transferred digitally to the hard drive of a computer (Apple Power MacIntosh, G4, Dual 500 MHz Power PC, 1 GB SDRAM, Apple Inc., Cupertino, CA, USA) for off-line evaluation. Magnification (460×) was achieved using long-distance objectives (Zeiss).

#### Microcirculatory analysis

Quantitative off-line analysis of the microscopy images was performed using the software package from the National Institute of Health (NIH Image 1.62) on a MacIntosh Computer (Apple Power Mac Dual 500 MHz G4 1 GB SDRAM, Apple, Cupertino, USA). Vascularization and effective vascular permeability of the striated muscle and bone were analyzed with a 20× long-distance objective in three different standardized ROIs: two in the border zone and one in the center of the chamber (Fig 2e and 2f). In the last ROI, measurement of the effective vascular permeability was performed.

Functional capillary density (i.e., the length of all perfused microvessels per observation area) was measured and is given in cm/cm^2^. Mean vessel diameters, centerline velocity, and volumetric blood flow were measured in all perfused microvessels in each ROI. Mean diameter was measured in μm perpendicularly to the vessel path. Centerline velocity was analyzed using the software package NIH Image 1.62 and expressed in μm/s. Volumetric blood flow was calculated as Q = π × (d/2)^2^ × v / 1.6 [pl/s], where 1.6 represents the Baker-Wayland factor to correct for the parabolic velocity profile in microvessels. Finally, the tissue perfusion rate was obtained using functional capillary density and volumetric blood flow in each ROI as described previously [12].

#### Effective vascular permeability

Effective vascular permeability was measured as described in detail previously [13–15]. Briefly, after i.v. administration of 0.1 ml of FITC-BSA (Molecular Probes, Invitrogen Lld., Paisley, UK), the fluorescence intensity was measured intermittently for 10 min and recorded digitally. The P-value was calculated as P = (1-HT)V/S{1/(I_0_-I_b_)*dI/dt+1/K}, where I was the average fluorescence intensity of the whole image, I_0_ the value of I after the filling of the vessels with FITC-BSA, and I_b_ the background fluorescence intensity. HT was the average hematocrit of vessels and is assumed to be equal to 19% [16]. V is the total volume and S is the surface area of vessels within the tissue volume covered by the surface image. The time constant of BSA plasma clearance K is 9.1 × 10^−5^ s [15].

### Experimental protocol

In this study, 28 NSG mice were divided into two groups, 14 for implantation of the dorsal skinfold chamber and 14 for implantation of the femur window. Group assignment was randomized. Five mice were lost to wound infection over the observation period. The microcirculatory values of these five mice that suffered infections were not included in the results, irrespective of the time they became infected. Intravital fluorescence microscopy analysis of vascularization, microhemodynamics, and effective vascular permeability was performed 7, 14, 21, and 28 days after chamber implantation. These time points were established in our previous studies [11, 17]. Preparation of the dorsal skinfold chamber and femur window were performed by a single physician. The off-line measurements from the video images were made by a single technician.

### Statistics

Differences between the study groups were analyzed using SPSS (IBM SPSS Statistics 19, Chicago, Illinois, USA) by the Mann-Whitney U Test. The Spearman rank correlation test was used to assess the correlation of statistics parameters. All values were expressed as means ± SEM (standard error of the mean). Statistical significance was based on p < 0.05.

## Results

Intravital fluorescence microscopy made it possible to study *in vivo* the microvasculature in striated muscle (dorsal skinfold chamber) and bone (femur window). The weight of the animals in both groups decreased slightly during the observation period, without reaching the criterion for exclusion of 15% body weight loss. All mean results are presented in Table 1.

**Table 1:** Microcirculatory parameters in bone and striated muscle tissue over the observation period.

Parameter	Group	Day 7	Day 14	Day 21	Day 28
**weight (g)**	femur window	29.08 ± 0.90	29.08 ± 0.51	28.17 ± 0.70	28.50 ± 0.79
	dorsal skinfold chamber	27.91 ± 1.03	26.82 ± 0.96	26.00 ± 0.71	26.09 ± 1.09
**diameter (μm)**	femur window	11.36 ± 0.46	11.60 ± 0.57	12.77 ± 0.88	14.64 ±1.06
	dorsal skinfold chamber	8.09 ± 0.29	8.31 ± 0.45	8.87 ± 0.62	8.34 ± 0.47
**velocity (μm/s)**	femur window	115.35 ± 6.79	135.16 ± 6.32	126.78 ± 4.74	120.98 ± 11.80
	dorsal skinfold chamber	122.40 ± 9.06	121.78 ± 6.22	109.17 ± 6.91	117.35 ± 6.96
**blood flow rate (μm**^**3**^**/s) x 10**^**3**^	femur window	12.87 ± 0.99	17.48 ± 1.99	19.00 ± 2.28	22.22 ± 2.38
	dorsal skinfold chamber	10.04 ± 1.84	9.02 ± 1.10	11.33 ± 2.42	8.64 ± 1.02
**vessel density (cm/cm**^**2**^**)**	femur window	137.00 ± 13.67	161.93 ± 8.42	145.84 ± 11.84	125.28 ± 13.93
	dorsal skinfold chamber	401.04 ± 28.06	348.32 ± 34.02	347.15 ± 30.02	363.20 ± 35.04
**tissue perfusion rate (ml/cm**^**2**^**/s) x 10**^**−5**^	femur window	31.63 ± 0.31	40.82 ± 0.36	40.75 ± 0.66	40.56 ± 0.64
	dorsal skinfold chamber	35.84 ± 0.63	33.98 ± 0.54	33.76 ± 0.52	34.93 ± 0.45
**permeability (cm/s) x 10**^**−3**^	femur window	5.42 ± 0.31	5.99 ± 0.49	6.24 ± 0.62	6.54 ± 0.57
	dorsal skinfold chamber	5.35 ± 1.01	3.61 ± 0.23	5.34 ± 0.72	3.75 ± 0.24

**Fig 3:**
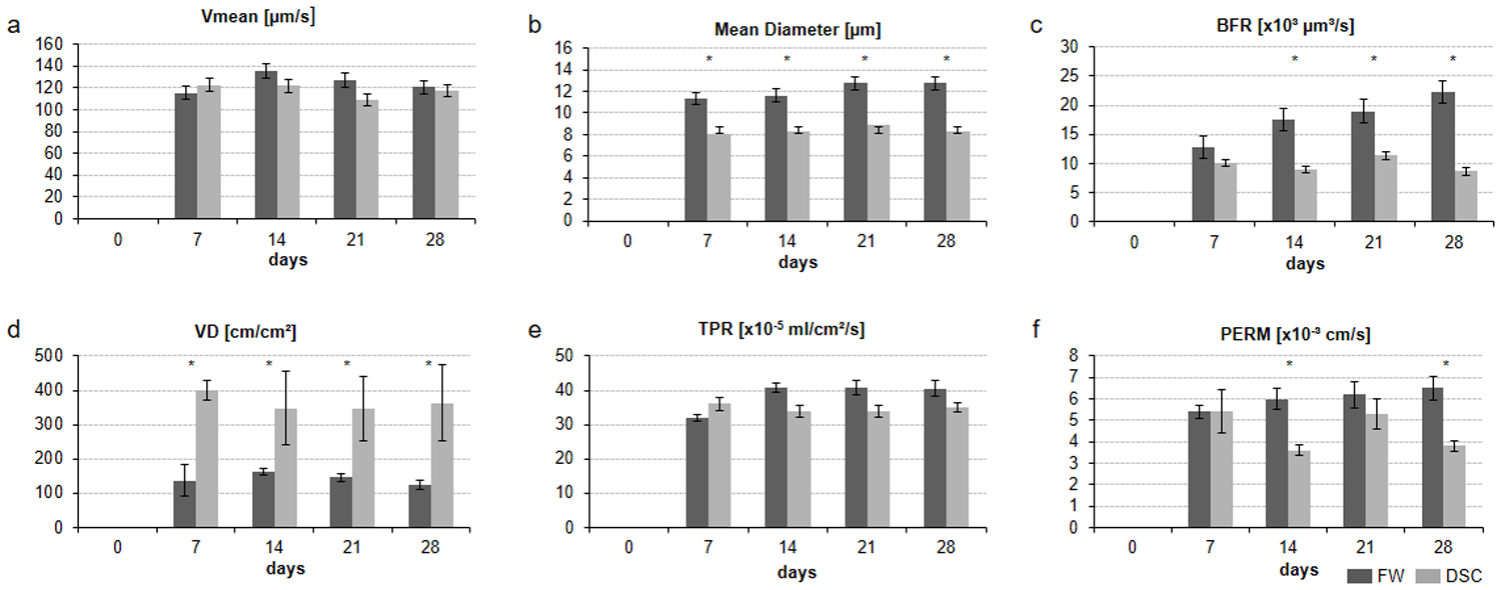
Microvascular parameters in the femur window group (dark gray bars) compared to the parameters in the dorsal skinfold chamber (light gray bars). All values are means ± SEM. Asterisks indicate p-values < 0.05. Abbreviations: Vmean, centerline velocity; BFR, blood perfusion rate; VD, vessel density; TPR, tissue perfusion rate; PERM, effective vascular permeability.

The centerline velocity did not differ significantly between the two groups during the observation period (Fig 3a). The vessel diameter was significantly lower in the dorsal skinfold chamber than in the femur window (7 days, p < 0.01; 14 days, p = 0.01; 21 days, p < 0.01; 28 days, p = 0.01) (Fig 3b).

The volumetric blood flow rate was higher in the femur window between days 14 (p = 0.01) and 28 (p = 0.01) than in the dorsal skinfold chamber (Fig 3c). During the entire duration of the observation period, the vessel density was significantly higher in the dorsal skinfold chamber than in the femur window (7 days, p < 0.01; 14 days, p = 0.01; 21 days, p < 0.01; 28 days, p < 0.01) (Fig 3d). The tissue perfusion rate, expressed as volumetric blood flow per time and area was similar in the two groups (Fig 3e).

The femur window group showed higher effective vascular permeability than the dorsal skinfold chamber group during the entire observation period, which reached statistical significance on days 14 (p < 0.01) and 28 (p = 0.01) (Fig 3f). The minimal data set of the microvascular parameters can be found in the S1 File.

## Discussion

In the present study we describe organ-specific differences, as well as similarities, in the microvascular network of striated muscle and bone *in vivo* in NSG mice over 28 days. We chose such a long observation period to guarantee reference values suitable for most subsequent studies, especially for tumor growth studies. To our best knowledge the presented baseline data on microvascular propertiers in bone and striated muscle of NSG mice *in vivo* are currently not available in the literature, despite the fact that these mouse strain is increasingly being used in biomedical research.

This severely immunocompromised mouse was described in detail in 2005 [2] and is used in cancer [18, 19], immune disease [20, 21], infectious disease [22, 23], hematologic disease [24, 25], stem cell [26], and diabetes [27, 28] studies. The NSG mouse provides an optimal environment for the engraftment of tumor cells and human cells, including stem cells [21, 29, 30]. Humanized mouse models can be used to compare tumor growth in immunocompromised NSG animals to tumor growth in animals with immune reconstitution (huPBL-NSG) generated from the same litter. The litter may allow a better assessment of tumor and immune system interactions [31].

Taken together, the previous studies of NSG mice are limited regarding the visualization of the microcirculation in striated muscle, bone, or the tumor microenvironment and often fail to reconstitute the complex architecture and physiology of multicellular tissues *in vivo*.

Intravital fluorescence microscopy is a powerful tool to study functional and morphological microvascular parameters in their natural environment [32]. However, these dynamic processes cannot be analyzed *in vitro* or *ex vivo* at the same ROI over long periods.

At present, a comparison of the physiological values of different mouse strains is unlikely to yield useful information because there have been few reports on the physiological microcirculatory parameters determined using either the femur window or dorsal skinfold chamber, as well as because of the considerable variability in instrumentation, protocols, anaesthetics, temperature, and operator experience. Hansen-Algenstaedt et al. analyzed microcirculatory parameters in the femur window by intravital fluorescence microscopy over 12 days in c57-black mice. We compared their values from days 6 and 12 with our values from days 7 and 14. Currently, there exist no femur window data with which to compare the data we obtained at exactly 1 and 2 weeks after surgery.

The c57-black mice of Hansen-Algenstaedt et al. exhibited lower vessel density, mean diamenter, volumetric blood flow, tissue perfusion rate and effective vascular permeability than our NSG mice but had a similar centerline velocity during the duration of the observation period. In conclusion, only the centerline velosity was similar between c57-black mice and NSG mice; the other microcirculatory parameters exhibited higher values in NSG mice, thereby revealing different microvascular network characteristics.

Although there have been many studies of the dorsal skinfold chamber, reports on the physiological microcirculatory parameters of striated muscle are rare. Lehr et al. [5] analyzed microcirculation in nude mice with dorsal skinfold chambers over 14 days with intravital fluorescence microscopy at days 3, 7, and 14. The data were given as mean values for the whole observation period. A comparison with our data shows that their mice had lower vessel density and mean diameter than our NSG mice but had a similar centerline velocity.

These comparisons suggest that NSG mice have different physiological microcirculatory parameters in bone and striated muscle than c57-black mice and nude mice, and thus, that reference values from c57-black mice and nude mice strains should not be used for experiments with NSG mice. However, direct head-to-head comparisons of these strains have not been made, making it impossible to rule out procedural bias.

Here, we describe the physiological microcirculatory parameters of bone and striated muscle in NSG mice to find organ-specific characteristics. When comparing the bone and striated muscle microcirculatory parameters in our study, we found that the vessel density was significantly higher in the dorsal skinfold chamber than in the femur window. In line with this observation, the number of vessels in striated muscle was higher, and the mean diameter lower, than in the femur window. The centerline velocity remained nearly constant during the observational period in both groups. The volumetric blood flow was calculated by using mean diameter and centerline velocity and was therefore significantly higher in the femur window than in the dorsal skinfold chamber. This observation may explain why tumors metastasize more often to bone than to striated muscle and will be essential for the proper analysis of data generated in future studies. Interestingly, there were no significant differences between the two groups in tissue perfusion rate, as indicated by the volumetric blood flow per time and area during the observation period. The effective vascular permeability in the femur window was higher than in the dorsal skinfold chamber during the observation period, and this reached statistical significance on days 14 and 28. This may be explained best by the difference in microvessel structure. In bone, the blood supply is guaranteed by a system of longitudinal canals (Haversian canals) connected by transverse canals (Volkmann’s canals), which are derived from periosteal and endosteal appositions [33, 34]. The dorsal skinfold chamber presents the usual striated muscle with regular microvessel anatomy [7]. Compared to previous studies with non-immunocompromised mice, the effective vascular permeability values reported here were significantly higher in both the femur window and dorsal skinfold chamber groups [9, 35]. Higher effective vascular permeability may be explained by endothelial dysfunction, which may be induced in NSG mice.

The study has several limitations. First, infection after chamber implantation is a critical problem of studies involving immunocompromised animals, as this might affect microvascular results. The dorsal skinfold chamber and femur window are very sensitive to infection; here, we lost five of our 28 mice to infection. Infections of these preparations often occur early after chamber implantation; in our case four animals between day 4 and 7 and one animal at day 14 after chamber implantation. Mice that suffered infections were associated with an obvious loss in chamber quality. The microcirculatory parameters are not measurable, and their prior measurements were excluded. We included only the remaining measurements of animals who survived the full 28 days in the results and, therefore, can rule out the influence of infection on bone and striated muscle microvasculature. Second, the use of analgesia is another confounder in interpreting the results of studies similar to ours. Metamizole typically used to reduce stress and pain for the animals post-operatively. Metamizole is the veterinarian-recommended analgesia for rodents, but its effects on microcirculation remain unclear.

## Conclusions

Here, we present site-specific differences between the femur and the striated muscle. Interestingly, the differences in morphologic and functional parameters resulted in similar tissue perfusion rates. Further studies are now needed to determine the impact of different mouse strains on organ perfusion, which might help elucidate the effect of differences in organ-specific perfusion on tumor growth.

## Supporting information

S1 File Minimal data set of microvascular parameters in NSG mice. (XLSX)
